# Crosstalk between RNA-binding proteins and non-coding RNAs in tumors: molecular mechanisms, and clinical significance

**DOI:** 10.7150/ijbs.109593

**Published:** 2025-04-21

**Authors:** Shiwen Cui, Qiu Peng, Qianfeng Ma, Xuemeng Xu, Wenlong Zhang, Xianjie Jiang, Shiming Tan, Wenjuan Yang, Yaqian Han, Linda Oyang, Shizhen Li, Jinguan Lin, Jiewen Wang, Longzheng Xia, Mingjing Peng, Nayiyuan Wu, Yanyan Tang, Qianjin Liao, Yujuan Zhou

**Affiliations:** 1 Hunan Key Laboratory of Cancer Metabolism, Hunan Cancer Hospital and the Affiliated Cancer Hospital of Xiangya School of Medicine, Central South University, Changsha, 410013, Hunan, China.; 2 Hunan Engineering Research Center of Tumor organoid Technology and application, Public Service Platform of Tumor organoids Technology, 283 Tongzipo Road, Changsha, 410013, Hunan, China.; 3 University of South China, Hengyang, 421001, Hunan, China.

**Keywords:** RNA-binding proteins, non-coding RNAs, phase separation, splicing, tumor immunity

## Abstract

RNA-binding proteins, integral in regulating RNA metabolism and gene expression, collaborate closely with non-coding RNAs, which are pivotal in post-transcriptional gene regulation. Both elements are essential for the progression of tumors. While recent research has increasingly illuminated their individual mechanisms, the intricate network interplay between them still requires further exploration. This article has provided a comprehensive review of the roles played by RNA-binding proteins and their associated non-coding RNAs in tumor biology. It delves into the intricate functions of various RNA-binding proteins in tumors, including their involvement in alternative splicing, m6A modification, alternative polyadenylation, and phase separation. Furthermore, it highlights the diverse and significant roles of different non-coding RNAs, such as microRNAs, long non-coding RNAs, and circRNAs, in tumor progression. The interaction between RNA-binding proteins and regulated non-coding RNAs is also explored, providing insights into their collective impact on metabolic reprogramming, immunity, drug resistance, metastasis, and ferroptosis. This in-depth exploration not only deepens our understanding of the mechanisms underlying tumorigenesis but also lays a foundation for developing innovative therapeutic strategies.

## Introduction

Tumors, a disease caused by genetic and epigenetic changes that cause cells to lose control over normal growth and differentiation, represent one of the most significant public health challenges today 1, 2. The progression of tumors involves a myriad of molecular mechanisms, with the regulation of gene expression being a crucial aspect. Gene expression goes through processes from transcription and translation to post-transcriptional and post-translational modifications, all of which are regulated at different levels 3, 4. Among them, RNA-binding proteins (RBPs) and non-coding RNAs (ncRNAs) are two types of molecules that play an important role at the gene transcription level, and there are complex interactions between them, which affect the biological behavior of tumor cells 5, 6. For example, RBPs can modulate the stability, transport, degradation, and functional performance of ncRNAs by recognizing and binding to their specific sequences or structures 7. Conversely, ncRNAs can also interact with RBPs to affect the expression, localization, activity, and phase separation of RBPs. These interactions take place in various cellular compartments, including the nucleus, cytoplasm, and cell membrane 8. Furthermore, these interactions manifest in a variety of physiological or pathological conditions, including hypoxia, inflammation, and tumor environments 9-11. As a result of these interactions, gene expression is finely regulated, thereby impacting tumor cell behaviors such as proliferation, migration, invasion, apoptosis, autophagy, differentiation, stemness, and immune evasion 12-16. For instance, recent studies have demonstrated that in metabolic disorders such as obesity-associated periodontitis, the long non-coding RNA AC018926.2 modulates the ITGA2/FAK/AKT signaling axis by binding to PARP1, thereby regulating stem cell differentiation 17.

This article aims to review the roles of RBPs and their associated ncRNAs in tumors, alongside the molecular mechanisms of their interactions. On one hand, the study of RBPs and ncRNAs has emerged as a highly active and promising research area in life sciences, encompassing not only the fundamental principles of gene expression regulation but also their significant implications for human health and disease 18. In gallbladder carcinoma, exosome-derived lncRNA TRPM2-AS activates the NOTCH1 signaling pathway through direct interaction with PABPC1, significantly enhancing tumor angiogenesis and metastatic dissemination 19. Particularly in oncology, RBPs and ncRNAs are identified as key players in numerous tumor-related signaling pathways and cellular processes, impacting critical aspects of tumor cell behavior such as proliferation, apoptosis, migration, invasion, and angiogenesis 20. On the other hand, gaining a comprehensive understanding of the roles and interaction mechanisms of RBPs and ncRNAs in tumors not only aids in elucidating the molecular mechanisms of tumor genesis and progression, but also facilitates the identification of novel targets and strategies for tumor diagnosis, prognosis, and therapy. Recent investigations have further revealed that circRNF13 stabilizes ITGB1 mRNA by enhancing the phase separation capacity of IGF2BP1, thereby promoting cisplatin resistance in oral squamous cell carcinoma 21.

Currently, there is considerable research on the roles of RBPs and ncRNAs in tumors, as well as their interaction mechanisms. However, most of these studies focus on one or a few classes of RBPs or ncRNAs, lacking a comprehensive and systematic overview of the field as a whole. In addition, these studies tend to ignore the interactions between RBPs and ncRNAs, focusing only on their respective functions. Hence, a comprehensive review of this field is necessary, aiming to analyze the roles and interaction mechanisms of RBPs and ncRNAs in tumors from multiple perspectives and levels, thereby offering a structured framework and guidance for future research.

Initially, we will discuss the functional attributes of various RBPs in tumors, encompassing aspects like alternative splicing, m6A modification, APA, and phase separation. Second, we will review the role of different types of ncRNAs in tumors and the interactions between RBPs and their regulated ncRNAs, including miRNAs, lncRNAs, and circRNAs. Subsequently, we will summarize the roles of RBPs and ncRNAs in tumor-related aspects such as metabolism, immunity, drug resistance, metastasis, and ferroptosis. Finally, we will look forward to future research directions and challenges in this field.

## Exploring the functional properties of RBPs in tumors

### Regulating of alternative splicing

Alternative splicing mediated by RBPs is a critical post-transcriptional regulatory mechanism that promotes mRNA stability and ensures protein diversity. In tumors, abnormal splicing events mainly include exon skipping (ES), alternative 5' splice site selection (A5SS), alternative 3' splice site selection (A3SS), mutually exclusive exons (MXE), and intron retention (RI) 22, 23. Among these, splicing abnormalities or splicing errors are a major factor in cancer development, and its occurrence is frequently linked to RBPs in tumor 24. RBPs are involved in alternative splicing in tumors in a variety of roles. Some RBPs are able to form complexes with splicing-associated core proteins that work together to control splicing molecules in tumor cells 25-28. Furthermore, certain RBPs modulate spliceosome activity by binding to the splicing sites 29, 30.

SR proteins, comprising 12 members (SRSF1-12) in mammals, share conserved domain architectures 31. These proteins regulate splice site selection and exon recognition by binding to pre-mRNA cis-elements and facilitating spliceosome assembly, thereby governing the production of alternative splice isoforms 32. Notably, SRSF10 exerts oncogenic functions through dual regulation of RNA splicing and metabolic reprogramming. In hepatocellular carcinoma (HCC), SRSF10 suppresses MDM4 exon skipping to downregulate p53 protein levels, concurrently reducing CD8+ T-cell infiltration, inhibiting IFNα/γ signaling, and inducing HIF1α-mediated PD-L1 upregulation, collectively driving tumor progression and immune evasion 33. In gliomas, SRSF10 serves as an independent prognostic marker by modulating BCLAF1 exon 5a splicing to dysregulate cell cycle progression, with its overexpression correlating with enhanced sensitivity to immune checkpoint therapy 34. Mechanistically, SRSF10 stabilizes MYB transcripts to upregulate glycolytic enzymes (GLUT1, HK1, LDHA), fostering lactate accumulation and histone H3K18 lactylation 35. This metabolic reprogramming establishes a feedforward loop that promotes M2 macrophage polarization and impairs CD8+ T-cell function, ultimately conferring resistance to anti-PD-1 therapy. Pharmacological inhibition of SRSF10 (e.g., compound 1C8) reverses immunosuppressive microenvironments and synergizes with immune checkpoint blockade, highlighting its potential as a pan-cancer therapeutic target 35.

Heterogeneous nuclear RNA (hnRNA) is the primary transcriptional product of RNA polymerase II in eukaryotes 36. Newly synthesized hnRNAs form complexes with various proteins in a non-selective manner during transcription, and heterogeneous nuclear ribonucleoproteins (hnRNPs) are indispensable protein components of these complexes 36. HnRNPs consist of RNA-binding domains (RBDs) and auxiliary domains, which guide hnRNPs to interact with target genes or other proteins by recognizing specific nucleic acid sequences. The hnRNP protein family includes several members, such as hnRNPH1, E1, A1, K, and PTBP1. Unlike SR proteins, the hnRNP family generally plays an inhibitory role in alternative splicing 37. SR proteins, such as SRSF1, typically promote spliceosome assembly and enhance exon inclusion by binding to exonic enhancers, whereas hnRNPs tend to bind to silencer elements, leading to exon skipping or intron retention through spatial hindrance, RNA structural remodeling, or competitive inhibition of SR protein activity. For example, hnRNPH1 regulates the alternative splicing of meiosis-related genes in germ cells by recruiting PTBP2 and SRSF3 38. Its loss results in chromosome synapsis defects and disrupted germ cell-support cell communication, ultimately leading to infertility. HnRNP E1, through binding to the nucleic acid structure of PNUTS pre-mRNA, inhibits its splicing to form lncRNA-PNUTS 39. When hnRNP E1 is silenced or undergoes nucleocytoplasmic shuttling, the inhibition is released, promoting the generation of oncogenic lncRNAs, which, in turn, enhance epithelial-mesenchymal transition (EMT) and breast cancer metastasis by sequestering miR-205. Additionally, PTBP1 (a member of the hnRNP family) accelerates exon skipping by forming intronic RNA loops or directly inhibits splicing activity through trans-exonic loops, thereby regulating splicing events of cancer-related genes 25. In apoptosis regulation, hnRNP A1 competes with SR protein SRSF1 for binding to Bcl-x pre-mRNA, inhibiting the splicing of the pro-apoptotic Bcl-xS isoform while promoting the expression of the anti-apoptotic Bcl-xL isoform, thereby enhancing tumor cell survival 37. This antagonistic regulation is particularly prominent in cancer, where the dynamic balance between hnRNPs and SR proteins determines the final outcome of splicing events, influencing tumor progression, metastasis, and chemoresistance.

The RNA Binding Motif Protein (RBM) family also plays a pivotal role in the regulation of alternative splicing. For instance, RBM22 is mainly involved in pre-mRNA splicing and is crucial for maintaining the conformation of the catalytic core of spliceosome, serving as a bridge between the catalytic core and other essential spliceosomal proteins 40. Additionally, RBM25 regulates alternative splicing by binding to the AMOTL1 pre-mRNA exon splicing enhancer motif 5'-CGGGCA-3' motif. This binding leads to increased expression of CircAMOTL1L, which in turn influences epithelial-mesenchymal transition (EMT) and suppresses the proliferation and metastasis of prostate cancer cells 41. In lung cancer cells, mutations in RBM10 have been identified as inducers of alternative splicing of key genes such as EIF4H, CD44, UBAP2L, and KDM6A, which ultimately promoting cell growth 42.

Additional regulators of alternative splicing orchestrate the occurrence of splicing events in cells. Notably, the knockdown of PRPF19 leads to the conversion of the MDM4 splice isoform from the stable full-length MDM4-fl to the unstable MDM4-s, which lacks exon 6 43. The splicing factor ESRP1 interacts with the exon flanking regions involved in forming Circ-BIRC6 through alternative splicing, thereby facilitating the formation of Circ-BIRC6 in human embryonic stem cells 44.

Recent studies have demonstrated that dysregulation of eIF4E can extensively reprogram alternative splicing in a mutation-independent manner. Specifically, eIF4E selectively promotes the nuclear export and translation of mRNAs encoding splicing factors (e.g., SF3B1 and U2AF1) via its nuclear RNA export activity, thereby markedly increasing the protein levels of spliceosomal components 45. Mechanistically, eIF4E directly binds to splicing factor transcripts (such as SF3B1 and U2AF1) and enhances their translational efficiency through polysome loading, while also physically interacting with the spliceosomal complex and specific precursor mRNAs to directly influence splice site selection. This dual action results in altered splicing patterns in approximately 800 transcripts in cell lines and around 4,600 transcripts in high-eIF4E acute myeloid leukemia (AML) patients. Further studies revealed that this process involves: (1) translation-splicing coupling regulation, whereby the RNA-binding protein HuR/ELAVL1 synergistically enhances splicing reprogramming by stabilizing splicing factor mRNAs 46; (2) the MNK-eIF4E signaling axis, wherein the oncogenic isoform MNK2b—generated by the selective splicing of MNK2—promotes splicing factor translation by sustaining eIF4E phosphorylation while circumventing p38-MAPK-mediated tumor suppression 47; and (3) an evolutionarily conserved mechanism, as evidenced by eIF4E's regulation of Sxl gene splicing in Drosophila, which affects sex determination 48. These findings reveal that eIF4E globally modulates the abundance of splicing factors and spliceosomal function, leading to widespread reprogramming of the splicing landscape that far exceeds the impact of single splicing factor mutations, thereby offering a novel mechanistic framework for splicing dysregulation in malignancies such as AML.

Given the crucial role of RBPs in alternative splicing, they have emerged as potential targets for therapeutic drugs. In recent years, scientists have developed various drugs that target RBPs or their mediated alternative splicing, including small molecule compounds and oligonucleotides 49. These drugs work by disrupting the interactions between RBPs and RNA or proteins, or by altering the expression level or activity of RBPs. This affects specific or a wide range of alternative splicing events, leading to therapeutic effects such as inhibition of tumor cell proliferation or induction of apoptosis. For instance, deletion of RBM10 increases the sensitivity of EGFR-mutated LUAD cells to spliceosome inhibitors. Combining spliceosome inhibitors with osimertinib, an EGFR tyrosine kinase inhibitor, enhances therapeutic efficacy, especially in RBM10-deficient LUAD, and overcome drug resistance 42. Therefore, a comprehensive understanding of the molecular mechanisms and networks by which RBPs regulate alternative splicing, as well as the development of drugs targeting RBPs or their mediated splicing events, is significant for clinical diagnosis and therapy 50.

### Regulation of alternative polyadenylation

**Figure 1:**
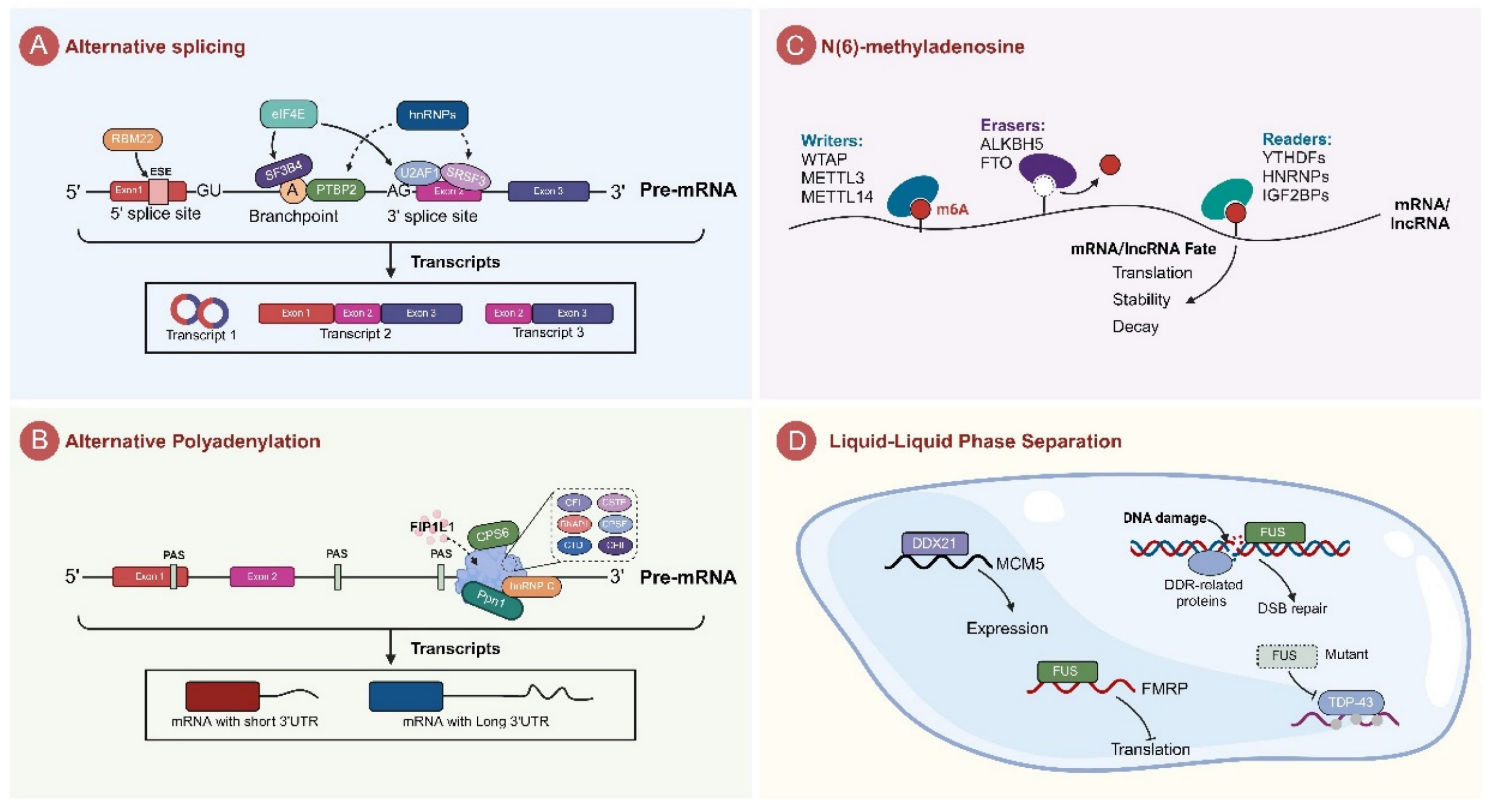
** Regulatory Functions of RNA-Binding Proteins in Tumor Biology. A.** RBPs and the spliceosome machinery synergistically regulate alternative splicing. **B.** The mechanism of APA regulation by RBPs is mainly through the regulation of the length of mRNA 3'UTRs. **C.** RBPs affects mRNA translation, stability, and degradation through dynamic regulation of N(6)-methyladenosine (m6A). **D.** RBPs perform biological functions through membrane-less organelles generated by liquid-liquid phase separation (LLPS).

Alternative polyadenylation (APA) is an RNA processing mechanism that generates transcripts with distinct 3' ends by selecting different polyadenylation sites within the mRNA's 3' untranslated region (3'UTR) or coding region 51, 52. This process regulates mRNA stability, translational efficiency, and protein function. Dysregulation of APA is widespread in cancer; for example, the prevalent shortening of the 3'UTR in cancer cells can lead to upregulation of oncogenes (such as NQO1) or inactivation of tumor suppressor genes, thereby promoting tumor proliferation, metastasis, and metabolic abnormalities 53-55. Recent findings indicate that various processing factors at the 3' end, along with RBPs like hnRNP C, CPSF6, and Ppn1 are significantly associated with the APA process 53, 56, 57. These proteins are usually involved in the APA process by interacting with complexes formed at the 3' end of pre-mRNAs, including CPSF, CSTF, CFI, CFII, CTD, and RNAPII (Fig.1B) 51. CPSF6, cleavage and polyadenylation specificity factor 6, is a vital member of the SR protein superfamily, whose structure and function are inextricably to the processing at the 3' end of mRNA. CPSF6 facilitates the use of proximal polyadenylation signals (PAS) by binding to the U-rich region on mRNA precursors. In hepatocellular carcinoma cells, CPSF6 significantly contributes to HCC progression by upregulating NQO1 expression through APA 53. Additionally, CPSF6 promotes the assembly of mRNA 3' end processing complexes and influences mRNA maturation and function by recruiting other processing factors like factor interacting with APOLA and CPSF1 (FIP1L1) 54. Interestingly, CPSF6 regulates APA in cancer cells through liquid-liquid phase separation (LLPS), independent of its expression level. This regulation leads to the shortening of the 3'UTR in cell cycle-related genes, thereby accelerating cell proliferation 54.

The hnRNP family includes a variety of proteins, such as hnRNP A1, A2/B1, C1/C2, H1, etc., which plays a multifaceted role in the regulation of alternative polyadenylation 58. For example, hnRNPC contributes to cancer progression in metastatic colon cancer cells by altering the selection of APA sites and affecting MTHFD1L and NAP1L1 expression 56. Furthermore, it is hypothesized that the shortening of the 3'UTR in lncRNA DSCAM-AS1 is mainly linked to the splicing factor hnRNPL 59. In addition, Hematopoietic- and neurologic-expressed sequence 1 (HN1) is associated with the senescence phenotype of cancer cells, and low expression of hnRNPA1 in cancer cells contributes to the prolongation of the 3'UTR on HN1, which regulates cancer cell senescence 55.

Other RBPs also contribute to gene expression regulation via APA. For instance, PPN1, a component of the DPS subcomplex, regulates the localization and stability of the CPSF complex in the nucleus through interactions with proteins like Dis2 and Swd22. This in turn affects the expression of phosphate homeostasis genes (e.g., pho1 and pho84) via an APA mechanism 57. Moreover, ELAV/Hu family proteins are known for regulating APA of pre-mRNAs in the drosophila nervous system, where they facilitate the extensive expression of mRNA isoforms with a long 3' UTRs 60.

In summary, APA represents a distinct type of alternative splicing, yet the precise mechanisms by which RBPs regulate APA remain an emerging area of research. Significantly, a growing number of studies have highlighted the impact of alternative polyadenylation on gene expression and disease progression 61. Consequently, deepening the understanding of the APA process has vital clinical implications and may help identify novel biomarkers and therapeutic targets.

### Regulation of m6A modification

In addition to alternative splicing, RBPs can also affect ncRNA function and expression through m6A modification 62. N6-methyladenosine (m6A) modification, a prevalent form of eukaryotic mRNA modification, comprises approximately 0.1%-0.4% of all adenosine residues in mRNAs 63. Modification of m6A involves three key components: the “writer” that add m6A modification to specific RNA sequences, the “eraser” that remove existing m6A modifications, and a “reader” that recognize and bind to m6A-modified RNA 64. The m6A modification exerts its functional effects by dynamically regulating RNA translation, stability, and processing.

The "writer" components (such as the METTL3/METTL14/WTAP complex) catalyze m6A modification. Methylation of targeted RNA modulates its secondary structure or binding sites, thereby affecting the recruitment of RNA-binding proteins (RBPs). For example, RBM10 can inhibit the m6A methylation of lncRNA MALAT1, a member of the lncRNA family, by recruiting METTL3. It also reduces MALAT1 expression by binding and regulating it, thereby affecting the phosphorylation of the downstream PI3K/AKT/mTOR pathway, and ultimately impacting the invasion and migration of NSCLC 65.

The "eraser" components (such as ALKBH5 and FTO) dynamically regulate m6A modification levels through demethylation. The removal of m6A modifications can directly impact RNA stability and processing efficiency. The m6A modification is continuously converted under the action of “writer” and “eraser”, dynamically regulating the transcription and translation of eukaryotic ncRNA. For instance, ALKBH5 can decrease the m6A methylation level of lncRNA-NEAT1, leading to the upregulation of NEAT1 expression. Elevated NEAT1 then acts as a scaffold, influencing the expression of EZH2 and consequently promoting the invasion and metastasis of gastric cancer (GC) cells 66. Furthermore, ALKBH5 demethylates pri-miR-194-2, an effect that relies on m6A modification, thereby inhibiting the biogenesis of miR-194-2 and consequently reducing distant metastasis of esophageal cancer. Additionally, ALKBH5 enhances the stability of circCCDC134 and increases its expression through demethylation of m6A modification, a process that subsequently facilitates the growth and metastasis of cervical cancer 67, 68.

The "reader" components (such as YTHDF1/2/3) regulate RNA fate by recognizing m6A modification sites. For example, YTHDF2 binds to m6A-modified RNAs and recruits the CCR4-NOT complex, thereby promoting the degradation of target RNAs 69; YTHDF1 enhances the translation efficiency of target RNAs by associating with ribosomal complexes; and the YTHDF family members (YTHDF1/2/3) competitively bind to the same target, thereby regulating its functional balance. For instance, in non-small cell lung cancer (NSCLC), YTHDF3 preferentially binds to the m6A sites on YAP pre-mRNA, while YTHDF1 and YTHDF2 competitively bind to respectively promote YAP translation or degradation, ultimately determining YAP protein levels and influencing tumor progression 68.

### Phase separation

RBPs coordinate the distribution and function of RNA within specific cellular regions, a process that is essential to ensure the timely and spatially accurate expression and functioning of RNA molecules 70. Consequently, the aggregation of specific molecules at precise cellular locations within membraneless organelles is essential for establishing order. Proteins that facilitate LLPS often possess intrinsically disordered regions (IDRs). These IDRs potentially mediate the initiation of LLPS through weak and non-specific affinity interactions with various targets 71.

RBPs have been shown to influence gene expression and function via LLPS 72, 73. For example, DDX21 is an RNA helicase containing a DEAD domain and is known to be involved in ribosomal RNA processing, RNA polymerase II-mediated transcription, and can affect gene expression regulation and cell differentiation, but the role of DDX21 in LLPS is still unclear. Consequently, Han et al. discovered that DDX21 forms a phase-separated condensate in colorectal cancer cells. This phase-separated DDX21 shows a high affinity for binding to the MCM5 gene locus, thereby modulating the expression of MCM5 (Fig.1D) 74. DDX3X and DDX3Y, encoded by genes on the X and Y chromosomes, are sexually dimorphic RNA helicases. Studies have shown that DDX3Y demonstrates a greater tendency for LLPS compared to DDX3X. This is attributed to variations in their N-terminal intrinsically disordered regions and ATPase activities. Such differences crucially influence mRNA translation repression and the aggregation of the FUS protein 75.

Furthermore, phase separation acts as a dynamic platform for responding to various cellular signals and stresses 76. Phase separation also facilitates the swift transport and distribution of RNA-binding proteins and RNA, thereby regulating RNA stability and availability. As an example, FUS, a protein involved in RNA metabolism and DNA repair, responds to DNA damage signals via phase separation. It forms a subnuclear foci at the DNA damage site and interacts with other DNA damage response (DDR) factors to promote the repair of DNA double-strand breaks (DSBs) 77. Additionally, the malfunction of FUS in amyotrophic lateral sclerosis (ALS) is associated with the development of this disease. Upon mutation of FUS, the protein accumulates in the cytoplasm and exhibits phase separation capabilities. This leads to the formation of LLPS condensates that disrupt the phase separation equilibrium of FMRP, thereby inhibiting protein translation 78.

Furthermore, RBPs can modulate the differential expression and function of RNA via the selective barrier created by phase separation. For instance, TDP-43 selectively binds to and regulates RNA containing long motif clusters via phase separation. Mutations in FUS can disrupt the phase separation equilibrium of TDP-43, leading to alterations in the aggregation number and size within TDP-43's binding regions and changes in TDP-43's RNA binding profile 79.

In summary, RBPs are pivotal in modulating the biological functions of RNA via phase separation mechanisms. Research in this field not only deepened our understanding of the mechanisms of intracellular RNA processing and expression, but also opened up avenues for novel therapeutic strategies. However, studies on the regulation of non-coding RNAs by RBPs through phase separation are still limited, and further exploration in this emerging field is necessary.

## The role of non-coding RNAs in tumors

### CircRNA

**Figure 2:**
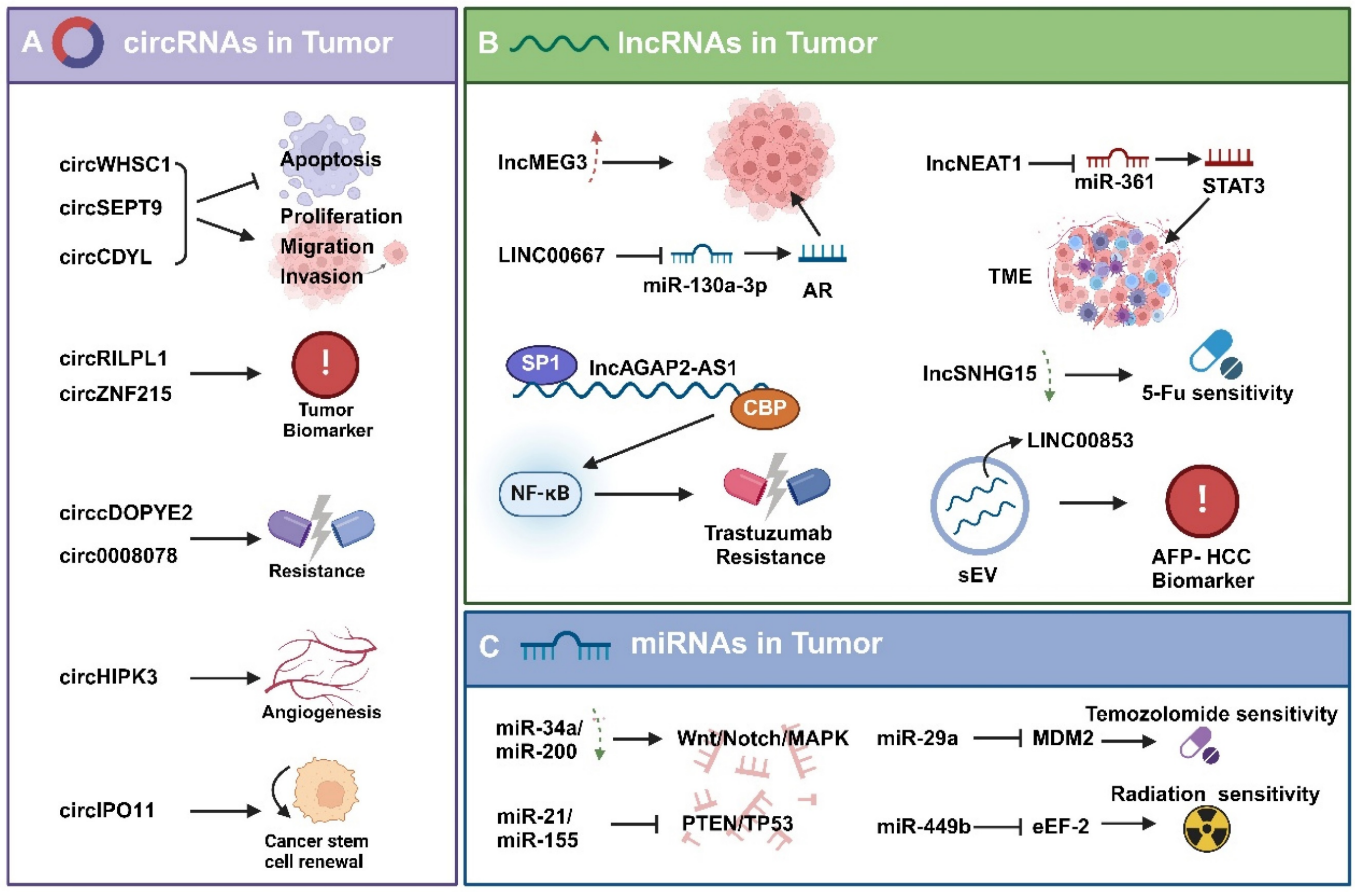
** Mode of action of non-coding RNAs in tumors. A**. circRNAs affect tumor cell apoptosis, proliferation and invasion, drug resistance, angiogenesis, stemness and other malignant progression. **B.** lncRNAs act in tumors through different mechanisms, including triggering apoptosis, regulating the tumor microenvironment (TME), affecting drug sensitivity, and serving as biomarkers. **C.** miRNAs in tumors influence drug sensitivity and radiosensitivity by regulating downstream signaling pathways.

Circular RNA (circRNA) is a novel type of non-coding RNA that is formed by the covalent closure of the 5' and 3' ends of the precursor RNA. They are mostly localized to the cytoplasm, are highly sequence conserved, are not easily degraded by nucleases, are very stable *in vivo*, and are specific to tissue or developmental stage. CircRNA is an important regulator of tumorigenesis and development, and recent studies have shown that circRNA can regulate the proliferation, apoptosis, migration and invasion of tumor cells 80. For example, circRNA WHSC1 is significantly overexpressed in uterine cancer tissues, which substantially enhances the proliferation, migration, and invasion capabilities of uterine cancer cells while concurrently reducing apoptosis (Fig.2A) 81. It is well known that elevated levels of circSEPT9 and circCDYL significantly boost the proliferation of tumor cells and simultaneously decrease apoptosis rates 82, 83. Moreover, circRNAs like circRILPL1 and circZNF215 hold promise as potential cancer biomarkers. Their expression levels in certain cancers are closely linked to disease severity and prognosis, making them useful tools for cancer diagnosis and prognostic 84, 85. Furthermore, certain circRNAs affect the responses of tumor cells to chemotherapeutic agents and play a role in tumor drug resistance by modulating drug metabolism or apoptosis pathways. As an example, the circRNA cDOPEY2 was found to be significantly down-regulated in cisplatin-resistant esophageal squamous cell carcinoma (ESCC) cells. The reintroduction of cDOPEY2 substantially improved the efficacy of cisplatin against ESCC cells 86. CircRNAs not only regulate intracellular processes but also play a significant role in modulating the tumor microenvironment. For instance, circRNA circHIPK3 is significantly upregulated in breast cancer, which contributes to tumor progression by affecting angiogenesis in endothelial cells in the tumor microenvironment 87. Additionally, circRNAs are closely related to cancer stem cells, which are crucial in tumor biology due to their ability to self-renewal and multi-directional differentiation. It has been shown that that the circRNA circIPO11 enhances the self-renewal ability of hepatocellular carcinoma stem cells, which in turn promotes their proliferation 88. It must be acknowledged that the study of circRNAs is still at an early stage and most of their mechanistic role in tumors remains elusive. Nevertheless, with technological advancements and deepening the understanding of circRNA functions, they demonstrate substantial potential for early diagnosis, prognosis and treatment of tumors.

### lncRNA

Long non-coding RNAs (lncRNAs) constitute a class of RNA molecules that do not encode for proteins and whose transcripts are more than 200 nucleotides in length. Initially, lncRNAs were considered as “transcriptional noise” because of their low sequence conservation, extremely low expression levels, and limited detectability in genetic screenings 89. In recent years, with the development of new technologies, an increasing number of studies uncovering the significant role of lncRNA in tumorigenesis, progression, prognosis, and treatment 90. For instance, the overexpression of lncRNA HOTAIR, which is commonly observed in various tumors including liver, breast, and lung cancers, is frequently associated with tumor progression, increased malignancy, and poor prognosis (Fig.2B) 91-93. Additionally, some lncRNAs function as tumor suppressors, such as lncRNA MEG3, whose diminished expression is closely associated with cancer progression and poor prognosis 94. LncRNAs are involved in tumor regulation via diverse mechanisms. One major way is to act as molecular sponges to absorb and modulate miRNA activity, which in turn influences the expression of miRNA target genes. For example, lncRNA LINC00667, which is overexpressed in liver cancer, acts as a molecular sponge for miR-130a-3p. This interaction exacerbates liver cancer progression by regulating androgen receptor expression 95. Secondly, lncRNAs can affect tumor cell activity by regulating protein function or localization through interaction with proteins. Particularly in breast cancer, SP1-induced overexpression of lncRNA AGAP2-AS1 upregulates MyD88 expression and activates the NF-κB signaling pathway by binding to the transcriptional coactivator CBP, which promotes breast cancer growth and enhances trastuzumab resistance 96. Additionally, certain lncRNAs play a role in regulating the tumor microenvironment. For instance, through the miR-361 regulatory network, lncRNA NEAT1 influences the expression of STAT3 and other genes pivotal to the tumor microenvironment, such as MEF2D, ROCK1, WNT7A, and VEGF-A, thereby altering the immune microenvironment of the tumor and facilitating the progression of aggressive endometrial cancer 97. LncRNAs also contribute to the development of tumor resistance. The down-regulation of lncRNA SNHG15 increases tumor cell sensitivity to 5-FU, whereas its overexpression promotes chemotherapeutic drug resistance 98. In terms of diagnosis and therapy, the expression patterns of certain lncRNAs are closely associated with tumorigenesis, progression and prognosis, and thus they may serve as effective cancer biomarkers. For example, the increased expression of lncRNA LINC00853 in serum small extracellular vesicles may serve as a novel biomarker for early-stage hepatocellular carcinoma, particularly in AFP-negative hepatocellular carcinoma (HCC) cases 99. Furthermore, therapeutic approaches targeting lncRNAs, including small interfering RNAs (siRNAs) and nucleic acid drugs, have demonstrated promise in cancer treatment. However, these strategies encountered significant challenges in terms of specificity, effective delivery, and tolerability 100.

### MiRNA

Micro RNAs (miRNAs) are a class of small non-coding RNAs, typically 19-24 nucleotides long, whose primary function is to regulate gene expression by inhibiting mRNA translation or inducing mRNA degradation through binding to the 3' untranslated region of mRNA. It is well-recognized that miRNAs have multiple biological functions. They can regulate key genes involved in cell proliferation, apoptosis, migration, and angiogenesis, thereby affecting cell growth, invasion, and metastasis. Furthermore, miRNAs play a significant role in the occurrence and progression of various diseases 101. The role of miRNAs in tumors is particularly noteworthy, and it is widely accepted that they have important regulatory roles in tumorigenesis and progression. Generally, miRNAs are classified into two types based on their effects on tumor progression: tumor-inhibitory miRNAs and tumor-promoting miRNAs 102. Tumor-inhibitory miRNAs play a pivotal role in hindering tumorigenesis and progression by specifically targeting and suppressing the activity of certain oncogenes or signaling pathways. For instance, the miR-34a and miR-200 families are often downregulated in various cancers. These miRNA classes specifically target and inhibit several critical tumor-promoting factors, including key components of signaling pathways like Notch, Wnt, and MAPK (Fig.2C). In this way, they effectively inhibit the proliferation, invasion, and migration of cancer cells 103, 104.

Conversely, the expression of certain miRNAs is elevated in tumors and are therefore classified as tumor-promoting miRNAs. For example, miR-21 and miR-155, are frequently upregulated in various cancers. They target and suppress key tumor suppressor genes such as PTEN and TP53, or elevate the expression of VEGF, which enhances angiogenesis and further promotes tumor development and progression 105, 106.

Recent studies have revealed that miRNAs not only regulate tumor development and progression, but also affect tumor response to treatment, particularly chemo-radiotherapy sensitivity. For instance, miR-29a can target the abnormal expression of MDM2, thereby enhancing the sensitivity of glioma cells to temozolomide treatment 107. Additionally, Studies have shown that elevated levels of miR-449b in tumors are positively correlated with the sensitivity of tumor cells to radiotherapy. Furthermore, eEF-2 kinase functions as a key intermediary in the radiation-sensitizing effect exerted by miR-449b 108. This finding highlights the potential of augmenting cancer therapy efficacy through the regulation of miRNA expression.

## The regulation of non-coding RNAs by RBPs in tumors

**Figure 3:**
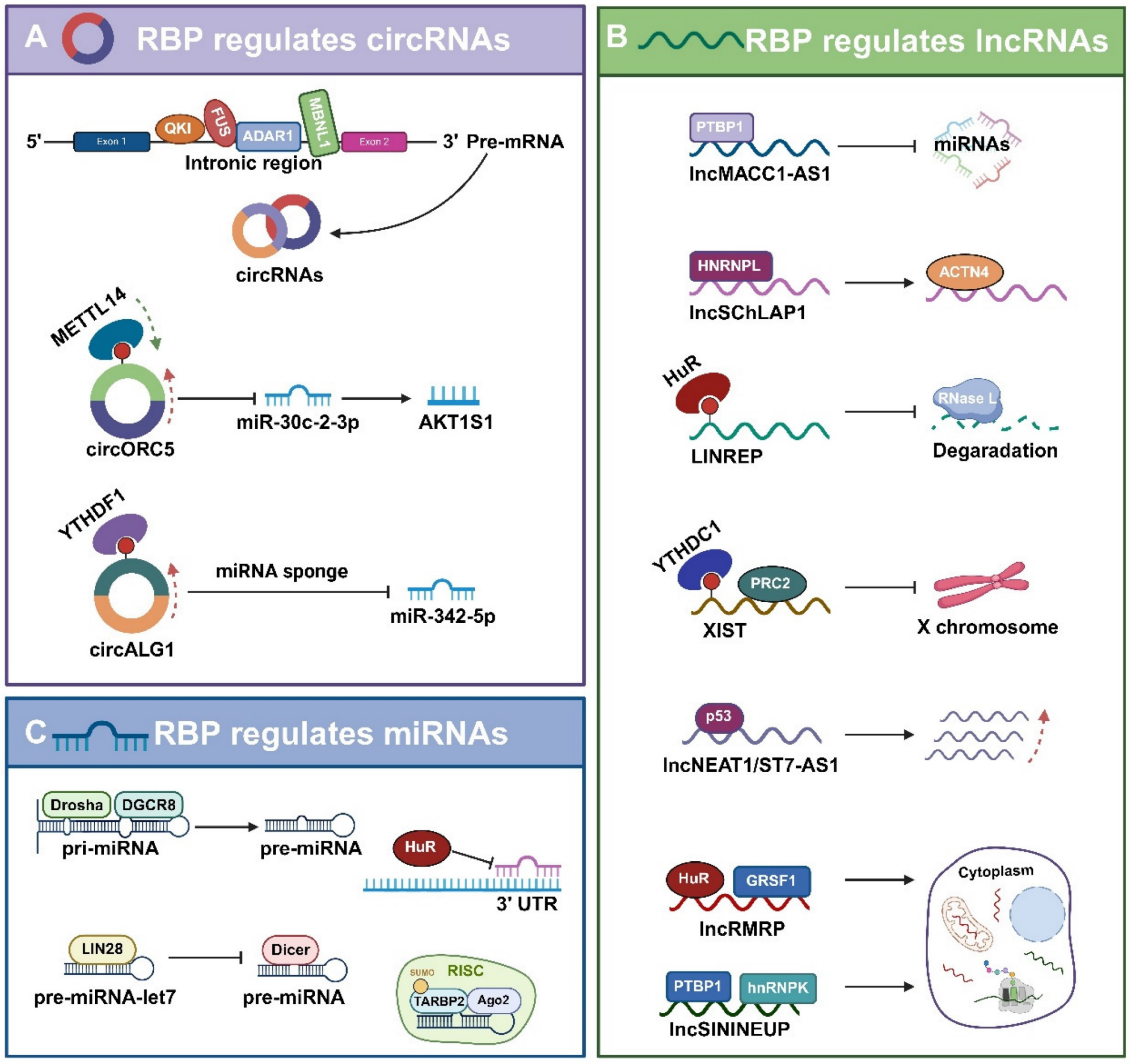
** The regulation of non-coding RNAs by RBPs in Tumors. A.** Some RBPs (e.g., QKI, FUS, ADAR1, and MBNL1) regulate the biogenesis of circRNAs, while others (e.g., METTL3 and YTHDF1) affect the stability of circRNAs.** B.** Some RBPs (e.g., PTBP1, hnRNPL, and HuR) control the stability and degradation of lncRNAs, others (e.g., YTHDC1 and p53) inhibit the transcription of lncRNAs, and some others (e.g., HuR, GRSF1, PTBP1, and hnRNPK) regulate the subcellular localization of lncRNAs.** C.** RBPs such as Drosha, DGCR8, Dicer, LIN28 bind to pri-miRNA/pre-mRNA and affect the processing and maturation of miRNAs; while RBPs such as Ago2, TARBP2, HuR bind to miRNA and affect their stability, translocation, and function.

Recent research highlights the significant role of RBPs in the biogenesis, functionality, and stability of non-coding RNAs, extending beyond their interaction with mRNA (Fig.3A).

### RBPs regulation of circRNAs

Despite circRNAs have fewer RBP-binding sites than their linear mRNA counterparts, there is strong evidence that they interact with RBPs, which play a crucial role in various aspects of circRNA biology, including their generation, post-transcriptional regulation, functional execution, translation, specific modifications, and potential involvement in extracellular transport pathways 80.

#### Roles of RBPs in the biogenesis of circRNAs

RBPs play an essential role in the regulation of circRNA production. For example, the quaking protein (QKI) significantly enhances circRNA production. QKI binds to specific RNA sequences in circRNA-producing precursor mRNAs and aligns these sequences closely, thus enabling splicing reactions between them. Specifically, QKI typically binds to intronic regions designated for reverse splicing. This binding acts as a “bridge” in circRNA generation, causing the RNA molecule to loop tightly enough for reverse splicing to occur 109-111. Besides QKI, numerous other RBPs, including FUS, ADAR1, MBNL1 have been recognized to have an effect on circRNA production 112-114.

#### RBPs regulate the stability of circRNAs

In circRNAs, m6A modifications are usually catalyzed by RNA-binding proteins, including METTL3, METTL14, and WTAP. For instance, METTL14 can inhibit the progression of gastric cancer by affecting the stability of circORC5 through m6A modification, which in turn affects the miR-30c-2-3p/AKT1S1 signaling axis. YTHDF1, as an m6A-modified “reader protein” enhances the stability of circALG1 and its binding affinity to miR-342-5p, thus significantly enhancing the function of circALG1 as a sponge. These proteins identify specific RNA sequences and introduce m6A modifications to them 115, 116.

### Roles of RBPs in the regulation of lncRNAs

RBPs regulates lncRNA stability, translocation, and transcription through various mechanisms such as alternative splicing, alternative polyadenylation, and m6A modification, thereby altering lncRNA function and expression (Fig.3B).

#### Roles of RBPs in the regulation of lncRNA stability

For instance, PTBP1 binds to lncRNA MACC1-AS1, thereby enhancing its sponging effect on miRNAs 117. Similarly, HNRNPL forms a stabilizing complex with lncRNA SChLAP1, which subsequently enhances the interaction of SChLAP1 with ACTN4 118. This binding to lncRNAs not only alters the stability of lncRNAs, but also affects their interactions with other molecules. LINREP, a long-stranded non-coding RNA that is prominently expressed in glioblastoma (GBM), has an m6A modification site and is recognized by HuR, a mechanism that protects it from RNase L degradation. When the m6A modification site of LINREP is mutated or the m6A-writing enzyme METTL3 is knocked down, both the stability and function of LINREP are affected 119.

#### Roles of RBPs in transcription and localization of lncRNAs

M6A modifications are also crucial in regulating the transcription of the lncRNA XIST, especially in the nucleus. For instance, YTHDC1, is a nuclear m6A “reader protein” that recognizes the m6A site on XIST and recruits the PRC2 complex to silence genes on the X chromosome 120. It has been observed that p53 acts directly on the promoters of lncRNAs, including NEAT1, whose transcription is regulated by the lncRNA ST7-AS1 121, 122.

RBPs can also influence the intracellular localization of lncRNAs. The RBPs HuR and GRSF1 can impact the stability and export of their target lncRNA RMRP, subsequently controlling RMRP's localization in the cytoplasm and mitochondria 123. Additionally, PTBP1 and hnRNPK can regulate the subcellular distribution of lncRNA SININEUP and the assembly of translation initiation complexes, thereby enhancing the translation of target mRNAs 124.

### Roles of RBPs in the regulation of miRNAs

RBPs can affect miRNA processing, stability, translocation, and function by binding to either miRNA precursors or mature forms (Fig.3C). The RBPs drosha and DGCR8 form a microprocessor complex that generates short-chain pre-miRNAs by binding to long-stranded pri-miRNAs 125. Furthermore, it has been shown that the efficiency of the activity of this complex varies depending on the type of miRNAs 126. Dicer, another key miRNA processing enzyme, cleaves pre-miRNAs into mature double-stranded miRNAs, where one strand is degraded and the other strand binds to the RNA-induced silencing complex (RISC) to fulfill miRNA functions 127. RBPs can also inhibit the miRNA maturation process. For instance, LIN28 is an RBP that is prominently expressed in embryonic development and stem cells and binds to the let-7 family of pre-miRNAs, thereby preventing cleavage by Dicer and inhibiting let-7 maturation 128.

RBPs also bind to mature miRNAs, thereby affecting their functional roles. HuR protein, which are commonly expressed member of the ElaV family, interacts with miRNAs, particularly inhibiting miRNA binding to the 3'UTR of mRNAs, and promoting the dissociation of miRISC from the target mRNAs, thereby diminishing the function of miRNAs 129. As previously mentioned, mature miRNAs processed by Dicer are incorporated into the RISC. Argonaute (Ago) protein, the core component of RISC, binds to mature miRNAs and recognizes the target mRNAs to inhibit their translation or degradation 101. TAR (HIV-1) RNA binding protein 2 (TARBP2) is also a component of RISC. SUMOylated TARBP2 undergoes a post-translational modification process that forms a RISC-loading complex by recruiting Ago2 and facilitates increased loading of pre-miRNAs into the RLC 130.

Collectively, RBPs play a crucial role in miRNA regulation, including binding to pri-miRNAs, pre-miRNAs, or mature miRNAs, as well as facilitating or inhibiting their processing, localization, stability, or targeting efficiency. These findings provide important insights into the miRNA regulatory network.

## Roles of non-coding RNAs regulated by RBP in tumors

### Tumor metastasis

**Figure 4:**
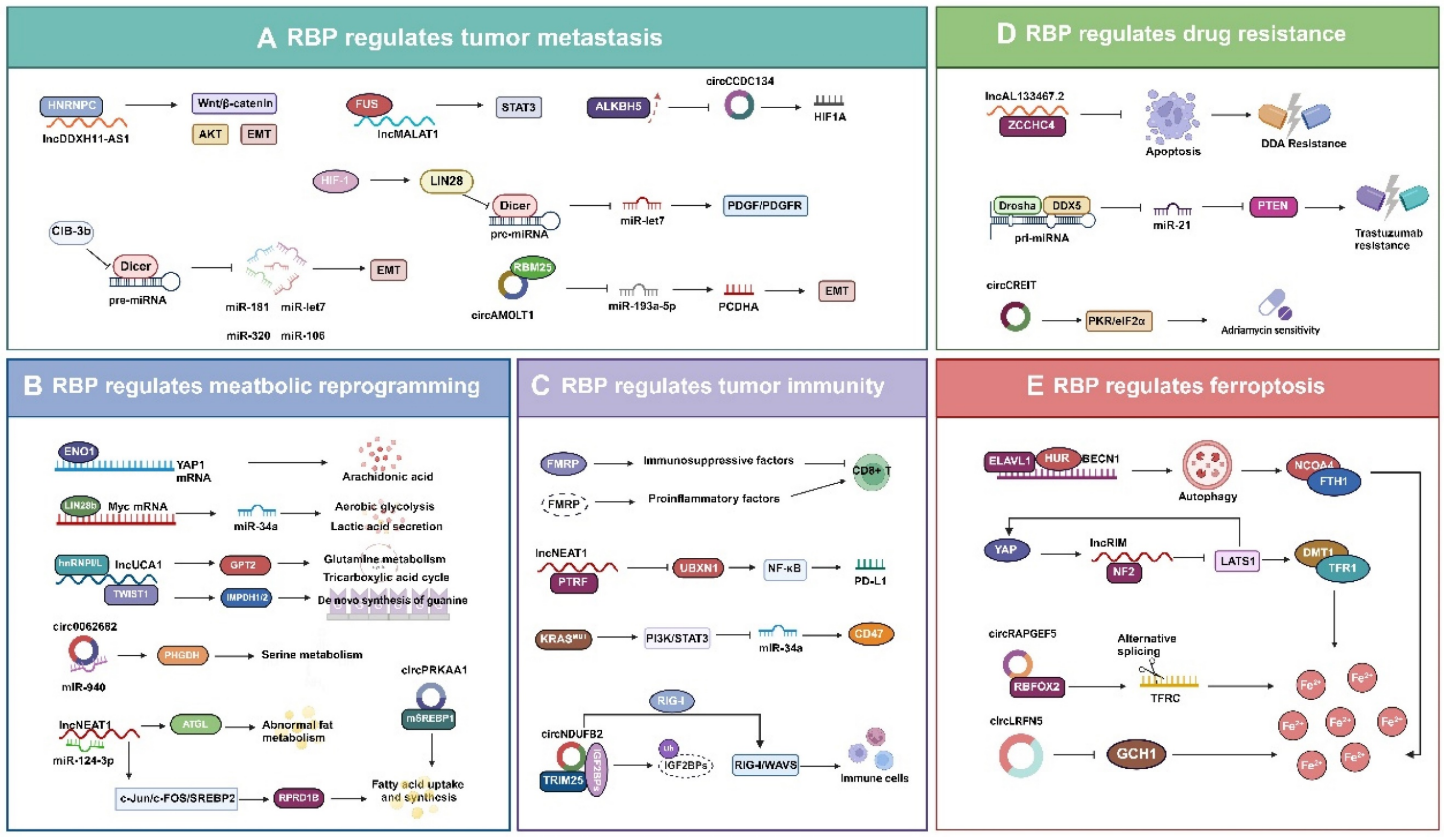
** Multifaceted roles of RBP-regulated non-coding RNAs in tumors. A.** RBPs promote the invasion and metastasis of tumor cells by affecting mRNAs and ncRNAs associated with cellular plasticity and regulating EMT and MET processes.** B.** RBPs modulate ncRNA to affect genes and enzyme activities related to metabolism, impacting various metabolic pathways such as glucose metabolism, amino acid metabolism, and fatty acid metabolism, influencing tumor progression. **C.** RBPs regulate the number of CD8+ T cells in the TME by directly controlling the activity of immunosuppressive and proinflammatory factors. Additionally, some RBPs regulate the transcription of ncRNAs affecting PD-L1 and CD47, suppressing the RIG-I-MAVS signaling pathway, thus facilitating tumor immune evasion.** D.** RBPs disrupt the conduction of related signaling pathways such as the apoptosis signaling pathway, PTEN pathway, and PKR/eIF2α pathway by affecting the expression levels of ncRNAs, regulating the drug sensitivity of tumor cells. **E.** RBPs and their regulated ncRNAs mainly affect the binding of ferroptosis-related molecules such as NCOA4 binding to FTH1, promoting the expression of ferroptosis-related genes DMT1 and TFR1, leading to excessive accumulation of iron ions, and ultimately inducing cell death by ferroptosis.

Tumor metastasis involves the process by which tumor cells detach from their primary site and colonize other parts of the body through the blood or lymphatic system, or directly invade adjacent tissues to form new tumors 131, 132. Regarding the pattern of tumor cell metastasis, Douglas Hanahan proposed the metastatic cascade model. The model suggests that tumor cell metastasis requires six steps: invasion, migration, traversal between vascular endothelial cells, circulation, colonization, and regrowth. These steps, in turn, are closely associated with factors like cellular plasticity, tumor heterogeneity, the tumor microenvironment, and the regulation of gene expression and signaling pathways 133. Current research is increasingly focusing on the interactions between tumor cells and their surrounding microenvironment, as well as on the epigenetic regulation of tumor cells, including the role of RBPs and their regulated ncRNAs 134, 135. During cancer metastasis, dysfunction of RBPs leads to aberrant expression or malfunction of mRNAs or ncRNAs linked to cellular plasticity, thereby impacting the epithelial-mesenchymal transition (EMT) and mesenchymal-epithelial transition (MET) processes in cancer cells 136-138. RBPs act as oncogenes or tumor suppressors by modulating the stability and processing of lncRNAs and circRNAs (Fig.4A). HNRNPC can specifically bind to the lncRNA DDX11-AS1 and promotes the Wnt/β-catenin and AKT pathways as well as the EMT process, thus facilitating glioma metastasis 139. The RBP FUS binds to the lncRNA MALAT1 and activates STAT3 signaling, which in turn promotes the proliferation and metastasis of lung cancer cells. RBM25 activates the EMT program in prostate cancer cell metastasis by inducing its biogenesis through binding to circAMOLT1 and subsequently regulating the circAMOTL1L/miR-193a-5p/Pcdha signaling pathway 41. The overexpression of ALKBH5 in cervical cancer decreases the expression and stability of circCCDC134, which in turn promotes the growth and metastatic of cancer cells by influencing HIF1A transcription 67. CIB-3b disrupts TRBP-Dicer interactions by binding to TRBP, leading to disrupted maturation of miRNAs (e.g., miR-181, miR-320, miR-106, and let-7) in hepatocellular carcinoma cells. This disruption affects EMT-associated signaling pathways and thus regulates metastasis 140. It has been demonstrated that HIF1 activates the expression of LIN28, which binds to the precursor of let-7 and prevents its cleavage by Dicer, thus inhibiting let-7 maturation. Increased activity of HIF1 and reduced expression of let-7 can enhance the invasion and migration of breast cancer cells, particularly in the case of brain metastasis. This correlation is evident in the modulation of the PDGF/PDGFR signaling pathway in breast cancer cells, which impairs the anti-metastatic effect of LK-99 against breast cancer 141. Overall, RBPs can promote or inhibit tumor cell metastasis by influencing the processing, stability, and subcellular localization of ncRNAs by regulating processes such as EMT and MET. RBPs can also modulate signaling pathways involved in growth, apoptosis, angiogenesis, and immune escape in tumor cells. Consequently, the role of RBPs and their regulated ncRNAs in tumor metastasis unveils new mechanisms and targets for cancer therapy and provide new perspectives and research avenues in the field of cancer biology.

### Metabolic reprogramming

One of the hallmarks of cancer is the reprogramming of energy production that favor increased glycolysis even in the presence of oxygen, a phenomenon known as the Warburg effect. This phenomenon, observed in various tumor types, coincides with the maintenance of the malignant phenotype of tumor cells through metabolic pathways involving fatty acids, cholesterol, and glutamine 142. RBPs and their regulated ncRNAs play a key role in the metabolic reprogramming of tumors, and can affect various metabolism-related pathways, including those of glucose, amino acids, and fatty acids, which can promote tumorigenesis and progression (Fig.4B).

Specific RBPs have been identified as regulators of glycolysis and glycolytic pathways. ENO1 protein is both a glycolytic enzyme enolase and an RNA-binding site, has been identified as an RNA-binding protein 143. ENO1 acts as an RBP that binds and stabilizes YAP1 mRNA, thereby promoting the growth of hepatocellular carcinoma cells by activating intracellular arachidonic acid metabolism 144. LIN28b enhances aerobic glycolysis and lactate secretion in tumor cells via the LIN28b/MYC/miR-34a pathway 145. In terms of amino acid metabolism, the interactions between these RBPs and ncRNAs can influence the synthesis and catabolism of essential amino acids in tumor cells, which may affect cell proliferation and survival 146. Besides upregulating GPT2 expression, LncRNA UCA1 also regulates inosine monophosphate dehydrogenase 1 and 2 (IMPDH1/2) expression via TWIST1, which alters metabolite levels and promotes guanine nucleotide de novo synthesis, thereby reprogramming the metabolism of bladder cancer cells 147. Circ_0062682 promotes PHGDH expression and activity by interacting with miR-940 to increase cellular serine metabolism and enhance proliferation, migration, invasion, and drug resistance in colorectal cancer cells 148. Moreover, RBPs and ncRNAs significantly regulate fatty acid metabolism, which in turn influences the energy homeostasis and signaling of tumor cells by modulating the synthesis and oxidation of fatty acids. Triglyceride lipase (ATGL) is a major enzyme in lipolysis and is regulated by NEAT1, which controls ATGL expression by interacting with miR-124-3p to modulate aberrant lipid metabolism and promote the proliferation of hepatocellular carcinoma cells 149. NEAT1 stabilizes the RPRD1B protein and enhances fatty acid uptake and synthesis via the c-Jun/c-Fos/SREBP1 signaling axis to promote primary tumor metastasis in lymph nodes 150. CircPRKAA1 binds to sterol regulatory elements binding protein 1 (mSREBP-1), which in turn increases fatty acid synthesis by upregulating its transcription, thereby promoting tumor growth 151. In summary, RNA-binding proteins and ncRNAs emerge as novel therapeutic targets due to their critical role in tumor metabolic reprogramming. The development of small molecule inhibitors or antagonists targeting molecules like ENO1, LIN28b, UCA1, and NEAT1 may be effective in inhibiting tumor growth and metastasis. Thus, exploring the mechanisms by which RBPs and ncRNAs contribute to the metabolic reprogramming of tumors remains a vital field of research in future cancer therapies.

### Tumor immunity

RBPs are crucial for the function of immune cells, mainly through the post-transcriptional regulation of RNA metabolism and function. The dysfunction of RBPs and aberrant RNA metabolism are closely associated with a variety of autoimmune and autoinflammatory diseases 152, 153. Various RBPs play pivotal roles in auto-reactive inflammatory responses by orchestrating a complex regulatory network of DNA, RNA, and proteins in immune cells (Fig.4C) 153.

Many human cancers have the ability to evade the adaptive immune system as certain cancer cells can achieve immune escape by expressing or modulating RBPs. These proteins affect the metabolism and function of self or viral-derived RNAs, thus inhibiting the expression or activity of immune checkpoint molecules and blocking the activation and destruction of immune cells 154, 155. FMRP is an RNA-binding protein that regulates the expression of various immune-related genes in tumor cells, thereby influencing the ability of tumor to evade immune surveillance. Elevated expression of FMRP in tumor cells promotes the secretion of immune-suppressive factors, including IL-33, Protein S, and exosomes, as well as the recruitment of regulatory T-cells and immune-suppressive macrophages 156. This would create an immune-suppressive tumor microenvironment, as well as depletion and clearance of cytotoxic CD8 T cells. Conversely, reduced or absent expression of FMRP in tumor cells leads to the secretion of pro-inflammatory factors, including CCL7, CCL9, CXCL9, and CXCL10, which recruits more cytotoxic CD8 T cells and enhances the anti-tumor response of lymphocytes 157.

Furthermore, ncRNAs regulated by RBPs play a crucial role in tumor immunity. NEAT1 enhances the expression of PTRF by interacting with the PTRF/Cavin-1, thereby stabilizing its mRNA, and PTRF activates NF-κB signaling by inhibiting the expression of UBXN1, thereby upregulating the transcription of PD-L1 and promoting the immune escape of tumor cells 158. In lung adenocarcinoma, mutations in KRAS can activate the PI3K-STAT3 signaling pathway, thereby inhibiting the expression of miR-34a and leading to elevated expression of CD47, which allows tumor cells to evade immune system surveillance 159.

CircNDUFB2 is lowly expressed in non-small cell lung cancer (NSCLC) and negatively correlates with the malignancy degree of NSCLC. CircNDUFB2 serves as a bridge to enhance the interaction between TRIM25 and IGF2BPs, subsequently promoting the ubiquitin-mediated degradation of IGF2BPs. Additionally, circNDUFB2 activates the RIG-I-MAVS pathway by being recognized by the RIG-I signaling mechanism, thereby recruiting immune cells into the tumor microenvironment and participating in the immune response of tumor cells 160.

### Drug resistance

Tumor cell resistance is a major obstacle to the treatment of patients with tumors, limiting the efficacy of chemotherapy, targeted therapy, radiotherapy, immunotherapy, and other treatments for a wide range of solid cancers. This resistance is also one of the leading causes of tumor-related deaths worldwide 161. Therapeutic resistance in cancer can be classified into natural and acquired resistance based on the timing of resistance development. Natural resistance that exists or rapidly develops in tumor cells before treatment may arise from genetic abnormalities, tumor heterogeneity, or intrinsic defense mechanisms. Conversely, acquired resistance which develops gradually in tumor cells post-treatment may result from modifications in driver oncogenes, activation of tumor-associated signaling pathways, or adaptation of the tumor microenvironment 162. Growing evidence indicates that epigenetic regulation plays a crucial role in tumor drug resistance, and interest in the mechanisms involving RBPs and their interacting is escalating 163. RBPs can influence the expression, function, and silencing of tumor-associated genes through various mechanisms, particularly by impacting ncRNAs, thus altering the drug response of tumor cells (Fig.4D). ZCCHC4 inhibits apoptotic signaling in hepatocellular carcinoma cells by interacting with lncRNA AL133467.2, thereby suppressing its pro-apoptotic function and thus promoting the chemoresistance to DNA-damaging agents (DDAs) in these cells 164. In 17q23-amplified breast cancer, the RNA-binding protein DDX5 interacts with the Drosha complex and affects the maturation of miR-21, which directly inhibits PTEN, an important mechanism for trastuzumab resistance in HER2+ breast cancers 165. Overexpression of circRNA-CREIT enhances the sensitivity of triple-negative breast cancer cells to adriamycin, which is linked to the inhibition of the PKR/eIF2α signaling axis 166. Numerous therapeutic strategies have been developed targeting RBPs and their regulated ncRNAs to overcome tumor resistance. Responsiveness to sorafenib treatment can be restored in sorafenib-resistant patient-derived xenograft mice by subcutaneous treatment with siRNAs targeting circRNA-SORE 167. The overexpression of circRNA17 in a mouse model, transplanted with enzalutamide-resistant cells demonstrated restoration of enzalutamide sensitivity in prostate cancer treatment 168. However, how to safely and effectively deliver siRNA or shRNA to the target site without side effects remains a major challenge in clinical applications.

### Ferroptosis

Ferroptosis is a mode of cell death induced by iron ions and lipid peroxidation that plays a significant role in tumorigenesis, progression, and metastasis. Ferroptosis is regulated through diverse cellular metabolic pathways, including redox homeostasis, iron metabolism, mitochondrial activity, and amino acids, lipids, and glucose metabolism 169. Recent evidence suggests that RBPs and their regulatory ncRNAs are instrumental in controlling key aspects of ferroptosis, including the glutathione-GPX4 pathway, glutamate/cystine transport, and the metabolism of both iron and lipids (Fig.4E) 170-172. ELAVL1/HuR interacts with BECN1 mRNA to stabilize its expression, thereby increasing autophagy levels and causing NCOA4 to bind to FTH1. This interaction results in the degradation of ferritin and an excessive release of iron ions, culminating in the ferroptosis of hepatic stellate cells 173. LncRIM is a lncRNA activated by YAP, which inhibits the kinase activity of LATS1 by binding to NF2. This inhibition enhances the transcriptional activity of YAP, promotes the expression of iron metabolism-related genes DMT1 and TFR1, and increases the level of intracellular iron ions. lncRIM also promotes its own expression by forming a feedback loop, thereby further enhancing YAP activity. This results in the excessive accumulation of iron ions, leading to lipid peroxidation and ultimately inducing ferroptosis in cells 174. The expression of lncRIM is regulated by various RBPs. HuR can bind to lncRIM and stabilizes its expression, thereby enhancing YAP activation. Conversely, some RBPs (e.g.YTHDF3) negatively regulate lncRNA GAS5, which reduces its activating effect on YAP 175, 176. Although the mechanism of circRNA role in ferroptosis remains unclear, it has been demonstrated that in endometrial cancer, circRAPGEF5 affects ferroptosis by regulating the alternative splicing of the transferrin receptor (TFRC) through interacting with the RNA-binding protein fox-1 homologue 2 (RBFOX2) 177. Furthermore, it has been demonstrated that circLRFN5 can cause elevated iron levels, increased lipid peroxidation, and the development of ferroptosis in GBM cells by reducing the expression of GCH1, a molecule that inhibits ferroptosis through the production of the antioxidant tetrahydrobiopterin (BH4) 178. Many studies have reported that miRNAs regulate the occurrence of ferroptosis by modulating genes involved in iron metabolism, antioxidants, and lipid metabolism 122, 179. Overall, although the mechanism of RBP-regulated ncRNAs in ferroptosis remains unclear, an increasing number of studies are aimed at unraveling this mystery. The role of RBPs and their regulated ncRNAs in ferroptosis is becoming increasingly significant.

## Conclusion and outlook

RBPs and their regulated ncRNAs play central roles in tumorigenesis and progression through mechanisms including alternative RNA splicing, m6A modification, APA processing, and phase separation 1, 18, 20. For instance, the RBP ZCCHC4 suppresses DNA damage-induced apoptosis by interacting with the long non-coding RNA AL133467.2, thereby promoting chemoresistance in hepatocellular carcinoma cells 164. Meanwhile, the ncRNA circRTN4 facilitates tumor growth and hepatic metastasis in pancreatic ductal adenocarcinoma via the circRTN4-miR-497-5p-HOTTIP axis while simultaneously stbilizing RAB11FIP1 to drive epithelial-mesenchymal transition 137. These findings not only elucidate the molecular basis of tumor heterogeneity but also highlight novel therapeutic opportunities targeting RNA regulatory networks.

Emerging studies have validated the therapeutic potential of targeting RBP/ncRNA interactions 180. Small-molecule inhibitors such as curaxin CBL0137 activate ZBP1 to induce necroptosis in cancer-associated fibroblasts and restore immune checkpoint blockade sensitivity in melanoma models 181. RNA-targeting therapies, including the miR-122 antagonist Miravirsen, have progressed to Phase II clinical trials 182. CRISPR/dCas13-based RNA editing platforms further enable precise modulation of oncogenic ncRNAs 183. Notably, serum exosomal miRNA panels (miR-181b, miR-193b, miR-195, miR-411) demonstrate clinical utility for preoperative prediction of lymph node metastasis in T1 colorectal cancer, with risk stratification models reducing overtreatment rates by 76% without compromising diagnostic accuracy 184, warranting further clinical validation.

Breakthroughs in combination therapies reveal synergistic effects between RNA-targeting agents and immunotherapies. The FMRP-targeting PROTAC degrader sc1-VHLL, delivered via lipid nanoparticles, specifically ubiquitinates FMRP in tumor cells, remodeling the tumor microenvironment by enhancing CD8+ T cell infiltration and reducing Treg populations in CT26-bearing mice, thereby converting immunologically "cold" tumors to "hot" lesions 185. Parallel studies show that ADAR1 ablation activates PKR/MDA5 pathways through reduced RNA editing, overcoming PD-1 resistance caused by antigen presentation defects 186. Additionally, radiotherapy-induced expansion of immunosuppressive MDSCs via the NF-κB-YTHDF2 feedback loop can be reversed by YTHDF2 inhibition, potentiating synergy with PD-L1 blockade. These findings collectively establish RNA processing proteins as pivotal regulators of immunomodulation and temporal therapeutic targets 187.

Future investigations should prioritize three directions: (1) mapping dynamic RBP-ncRNA interactomes across tumor subclones using spatial transcriptomics and RNA *in situ* imaging; (2) optimizing RNA structure-targeted drug design through AI-driven screening platforms; (3) accelerating clinical translation via interdisciplinary frameworks integrating organoid models and PDX platforms. As the RNA epigenetics landscape becomes increasingly deciphered, RBP/ncRNA-centered therapeutic paradigms promise to redefine precision oncology.

## Abbreviations

RBP: RNA-binding protein
ncRNA: non-coding RNA
AS: Alternative splicing
PPT: polypyrimidine tract
L-3′ SS: 3′ splice site of exon 3a
S-3′ SS: original 3′ splice site of exon 3
AML: acute myeloid leukemia
SR proteins: serine/arginine-rich proteins
SF3B1: Splicing factor 3b subunit 1
U2AF1: U2 small nuclear RNA auxiliary factor 1
SF3B4: Splicing factor 3b subunit 4
hTERT: human telomerase reverse transcriptase
NSCLC: non-small cell lung cancer
DR8: Death receptor 8
hnRNP: heterogeneous nuclear ribonucleoprotein
SE: skipped exons
A5SS: alternative 5' splice site selection
A3SS: alternative 3' splice site selection
MXE: mutually exclusive exons
RI: intron retention
PTBP2: Polypyrimidine tract-binding protein 2
SRSF3: Serine/arginine-rich splicing factor 3
RBM: RNA binding motif protein
EMT: epithelial-mesenchymal transition
EIF4H: Eukaryotic translation initiation factor 4H
CD44: Cluster of Differentiation 44
UBAP2L: Ubiquitin-associated protein 2-like
KDM6A: Lysine demethylase 6A
PRPF19: Pre-mRNA processing factor 19
MDM4: MDM4 regulator of p53
ESRP1: Epithelial splicing regulatory protein 1
EGFR: Epidermal growth factor receptor
LUAD: Lung adenocarcinoma
APA: Alternative Polyadenylation
3'UTR: 3' untranslated region
hnRNP C: Heterogeneous nuclear ribonucleoprotein C
CPSF6: Cleavage and polyadenylation specificity factor subunit 6
Ppn1: Protein phosphatase 1
CPSF: Cleavage and polyadenylation specificity factor
CSTF: Cleavage stimulation factor
CFI: Cleavage factor I
CFII: Cleavage factor II
CTD: C-terminal domain
RNAPII: RNA polymerase II
PAS: polyadenylation signals
NQO1: NAD (P)H quinone dehydrogenase 1
FIP1L1: Factor interacting with APOLA and CPSF1
LLPS: liquid-liquid phase separation
MTHFD1L: Methylenetetrahydrofolate dehydrogenase 1-like
NAP1L1: Nucleosome assembly protein 1-like 1
HN1: Hematopoietic- and neurologic-expressed sequence 1
DPS: Developmental pluripotency associated 2
Dis2: Dis2 phosphatase
Swd22: Snf2-related CREBBP activator protein
ELAV/Hu: ELAV like RNA binding protein/Hu protein
m6A: N6-methyladenosine
WTAP: Wilms tumor 1 associated protein
METTL: Methyltransferase like
PI3K: Phosphoinositide 3-kinase
AKT: AKT serine/threonine kinase
mTOR: Mechanistic target of rapamycin kinase
ALKBH: AlkB homolog
FTO: Fat mass and obesity-associated protein
EZH2: Enhancer of zeste 2 polycomb repressive complex 2 subunit
YTHDF: YTH N6-methyladenosine RNA binding protein
YAP: Yes-associated protein
IDRs: intrinsically disordered regions
DDR: DNA damage response
DSBs: DNA double-strand breaks
ALS: amyotrophic lateral sclerosis
circRNA: Circular RNA
ESCC: esophageal squamous cell carcinoma
LncRNA: Long non-coding RNAs
NF-κB: Nuclear factor kappa-light-chain-enhancer of activated B cells
HCC: hepatocellular carcinoma
siRNAs: small interfering RNA
miRNA: Micro RNA
QKI: quaking protein
HNRNPL: Heterogeneous nuclear ribonucleoprotein L
PRC2: Polycomb repressive complex 2
HuR: Human antigen R
GRSF1: G-rich RNA sequence binding factor 1
DGCR8: DiGeorge syndrome critical region gene 8
RISC: RNA-induced silencing complex
Ago: Argonaute
TARBP2: TAR (HIV-1) RNA binding protein 2
SUMO: Small Ubiquitin-like Modifier
RLC: RISC Loading Complex
HIF1: Hypoxia-inducible factor 1
PDGF/PDGFR: Platelet-derived growth factor/Platelet-derived growth factor receptor
ENO1: Enolase 1
LIN28b: Lin-28 Homolog B
IMPDH1/2: Inosine monophosphate dehydrogenase 1 and 2
TWIST1: Twist family bHLH transcription factor 1
ATGL: Adipose triglyceride lipase
mSREBP-1: Membrane-bound sterol regulatory element-binding protein 1
KRAS: Kirsten rat sarcoma virus
IGF2BP: Insulin-like growth factor 2 mRNA binding protein
DDA: DNA-damaging agents
shRNA: Short hairpin RNA
GPX4: Glutathione peroxidase 4
ELAVL1: ELAV like RNA binding protein 1
HuR: Human antigen R
NCOA4: Nuclear receptor coactivator 4
FTH1: Ferritin heavy chain 1
NF2: Neurofibromin 2
DMT1: Divalent metal transporter 1
TFR1: Transferrin receptor 1
TFRC: Transferrin receptor
RBFOX2: RNA-binding protein fox-1 homologue 2
GBM: Glioblastoma multiforme
BH4: Tetrahydrobiopterin
